# Cognitive improvement of compound danshen in an Aβ25-35 peptide-induced rat model of Alzheimer’s disease

**DOI:** 10.1186/s12906-015-0906-y

**Published:** 2015-10-23

**Authors:** Min Liu, Haibiao Guo, Chuyuan Li, Deqin Wang, Jingang Wu, Canmao Wang, Jiangping Xu, Ren-an Qin

**Affiliations:** Modern Chinese Medicine Research Institute of Hutchison Whampoa Guangzhou Baiyunshan Chinese Medicine Co., Ltd., Guangzhou, 510515 Guangdong Province China; Department of Pharmacology, School of Pharmaceutical Sciences, Southern Medical University, Guangzhou, 510515 Guangdong Province China; Post Doctoral Scientific Research Center, Guangzhou Pharmaceutical Corporation, Guangzhou, 510515 Guangdong Province China

**Keywords:** Alzheimer’s disease, Compound danshen, Insulin-degrading enzyme (IDE), Neprilysin (NEP)

## Abstract

**Background:**

Senile dementia mainly includes Alzheimer' s disease (AD) and vascular dementia (VD). AD is a progressive and irreversible neurodegenerative disorder that is accompanied with a great deal of social burden. The aim of this study was to investigate the effect of Compound Danshen (CDS) on learning and memory of alzheimer’s disease (AD) rat model, as well as to explore the possible connection between CDS and the associated molecules of amyloid beta (Aβ).

**Methods:**

Rats were injected with Aβ25–35 peptide intracerebroventricularly and CDS were subsequently administered once daily for 23 days. Rats’ behavior was monitored using Morris water maze and passive avoidance. Real time PCR and Western blotting were used in determining amyloid precursor protein (APP), β-site APP cleaved enzyme-1(BACE1), Presenilin-1 (PS1), Insulin-degrading enzyme (IDE) and neprilysin (NEP) in hippocampus.

**Results:**

The AD model group presented with spatial learning and memory impairments. CDS and donepezil administration significantly ameliorated the Aβ_25–35_ peptide-induced memory impairment in both Morris water maze (*P* < 0.05) and passive avoidance task (*P* < 0.01) compared to the AD model group. Real time PCR results suggested that CDS significantly decreased APP mRNA, PS1 mRNA and increased IDE and NEP mRNA levels. Western blotting analyses showed that CDS decreased the protein expression of APP and PS1 and increased IDE expression.

**Conclusion:**

CDS improved spatial learning and memory by down-regulating APP, PS1 levels and up-regulating IDE. In future, CDS may have significant therapeutic potential in the treatment of AD patients.

## Background

Senile dementia mainly includes Alzheimer’s disease (AD) and vascular dementia (VD). AD is a progressive and irreversible neurodegenerative disorder that is accompanied with a great deal of social burden. The accumulation of amyloid-beta (Aβ) in the brain may directly contribute to the degeneration of neurons during the pathogenesis of AD [1]. Therefore, Aβ protein is considered to be a direct cause of AD and is a target for AD treatment. Acetylcholinesterase inhibitors are prescribed to AD [2]. However, these drugs have limited effects on AD [3]. Accordingly, drugs to prevent the onset of AD and its progression still need to be explored. In recent years, studies have further suggested that the steady levels of Aβ peptide in the brain is regulated by the rate of production of amyloid precursor protein (APP) via β- and γ-secretases and degradation by the activity of several enzymes. Therefore, an inhibition of Aβ production and an increase in its removal could be a novel strategy for AD. Production of Aβ was determined by the activity of β-secrectases and γ-secretases, whereas several enzymes including neprilysin (NEP) and insulin-degrading enzymes (IDE) are capable of removing Aβ peptides [4, 5].

It appears that accumulation of Aβ can theoretically occur because of insufficient clearance of Aβ [6]. Recently, NEP and IDE have been studied most extensively. NEP is a membrane-bound zinc-dependent metalloprotease that degrades a number of peptides both *in vitro* and *in vivo* [7]. Similar to NEP, genetic deletion of IDE in mice increases cerebral Aβ levels [8]. Their critical role in neurodegenerative disease has been demonstrated in NEP- and IDE-knockout animals [9].

Compound Danshen (CDS) is composed of Danshen (Radix *Salviae Miltiorrhizae*), Sanqi (Radix *Notoginseng*), and Bingpian (*Borneolum*). CDS is indicated to be widely used for heart diseases and angina pectoris [10, 11]. Interestingly, our previous study has demonstrated that CDS was able to improve learning and memory in the AD model rats and lower the production of β-amyloid precursor proteins [12, 14], as well as decreased the amount of the excitatory amino acid glutamate in the brain [11]. In addition, CDS also reduced the APP mRNA expression in the transgenic cell model of AD [15]. However, whether CDS has an impact on Aβ degrading enzymes remains to be elucidated. In the present study, to further examine the mechanisms of CDS-mediated cognitive improvement in Aβ_25–35_ peptide-induced rat model, we performed behavioral studies, Real time PCR test and Western blotting analyses.

## Methods

### Animals

Fifty-four Male Sprague–Dawley rats (2 months old, 300–350 g), supplied by the Laboratory Animal Center of the Southern Medical University (Certificate No. 20110015 by the Guangdong Medical Laboratory Animal Administration Committee), were used for the experiment. The animals were housed in an air-conditioned room (21 °C ± 2 °C) under a 12-h light/dark cycle (lights on 07:00–19:00), with food and water freely available. All experiments were carried out in accordance with the NIH guide for the care and use of laboratory animals. The procedures were approved by the Animal Care and Use Committee of the Southern Medical University.

### Drugs and reagents

Donepezil hydrochloride (Eisai China Inc.) was directly dissolved in sterile distilled water. CDS extract, encompassed danshen, sanqi and bingpian in proportions of 450:141:8, was provided by Guangzhou Baiyunshan Hutchison Whampoa Chinese Medicine Co.Ltd. The compound danshen was prepared as previous study [10]. Briefly, Salvia miltiorrhiza (450 g), Notoginseng (141 g) and Borneolum Syntheticum (8 g), were extracted with 4.8 L ethanol heating reflux for 1.5 h, filtered, ethanol were recycled and filtered solution were concentrated to moderate volume; then the dregs were extracted with 4.8 L 50 % ethanol heating reflux for 1.5 h, filtered, ethanol were recycled and filtrate were concentrated to moderate volume; dregs were extracted with 3.6 L water for 2 h, filtered, filtrate were concentrated to moderate volume; *Radix Notoginseng* were crushed into fine powder, combined with the above concentrate solution and the moderate auxiliary materials made into grain, dried; *Borneolum Syntheticum* were porphyrized, blending with the grain. It was dissolved in sterile distilled water. And both mixtures were stored at 4 °C for use.

Aβ_25–35_ (Sigma-Aldrich, St. Louis, Missouri) was dissolved in sterile distilled water at a concentration of 1 mg/ml as a stocking solution. Aβ_25–35_ was “aged” by incubation at 37 °C for 4 days and stored at −20 °C for use [16, 17].

### Intracerebroventricular injection of the Aβ_25–35_ peptide and drug administration

Rats were anesthetized by intraperitoneal injections of 10 % chloralhydrate at a dose of 300 mg/kg and placed in a stereotaxic holder. A midline sagittal incision was made in the scalp and two holes were drilled in the skull over the lateral ventricles using the following coordinates: 0.8 mm posterior to bregma and 1.5 mm lateral to the midline. All injections were given using a 10-μl Hamilton syringe equipped with a 26-gauge needle. The dura was perforated with the needle of the microsyringe, which was inserted 4.0 mm beneath the dura. Animals were infused with 5 μl/side of sterile distilled water (control), aggregated Aβ_25–35_ (2 μg/μl), into bilateral cerebral lateral ventricles at a rate of 1 μl/min; the needle was left in place for 5 min. Then, the needles were removed and rats were kept on a warm pad until they were awakened. The dose of Aβ_25–35_ used in this study was based on our previous study [18]. To determine the neuroprotective effect on AD rats, the Aβ_25–35_ treated rats were treated with CDS of different doses and donepezil once daily for 23 days (including duration of behavior test).

**Fig. 1:**
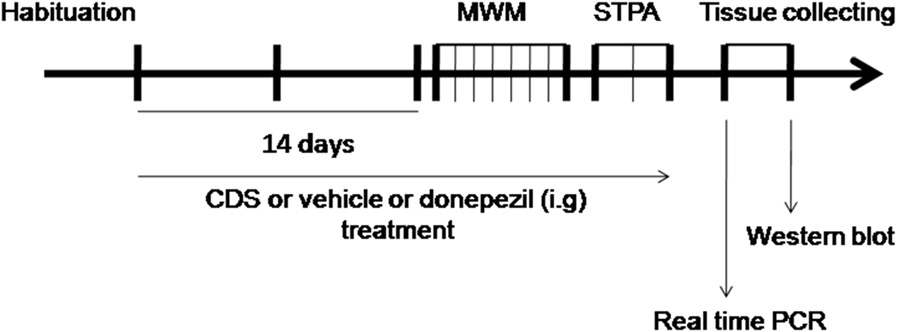
Schematic timeline of drug treatment and behavioral test order in rats. After 1 week of habituation in the animal room, rats were subjected to donepezil or vehicle or CDS treatment for 2 weeks once daily followed by behavioral tests. At the end of the tests, all the rats were decapitated for collection of brain tissue. MWM, Morris water maze; STPA, Step-through passive avoidance

Experiment was performed to test the effect of CDS on Aβ_25–35_-induced memory impairment using Morris water-maze and step-through passive avoidance tasks. Specifically, all of the rats were randomly divided into 6 groups for the experiment: (a) Vehicle 1 (for Aβ_25–35_) + vehicle 2 (for CDS and donepezil), (b) Aβ_25–35_ + vehicle 2, (c) Aβ_25–35_ + CDS (130 mg/kg), (d) Aβ_25–35_ + CDS (260 mg/kg), (e) Aβ_25–35_ + CDS (520 mg/kg), (f) Aβ_25–35_ + donepezil (0.5 mg/kg). One day after cerebroventricular microinfusions of Aβ_25–35_ (10 μg/side) or its vehicle, rats were treated (i.g.) with CDS or donepezil or vehicle 2, once daily for 14 days prior to the beginning of Morris water maze, followed by passive avoidance task (Fig. 1). The doses of CDS selected in present study were based on our previous report [14].

### The Morris water maze test

Morris water maze (MWM) was carried out for 14 days following the microinjection [18]. A circular pool (120 cm in diameter and 45 cm high), was filled with water (21 °C; 37.5 cm deep) and was made opaque with black ink and was divided into four equally spaced quadrants (north, south, east, and west). A transparent platform (10 cm in diameter) was placed at the east quadrant, 40 cm from the wall, with its surface 2 cm below the surface of the water. Acquisition training were performed with rats twice a day for 5 consecutive days with the interval of 3 h. During each trial, the rat were placed in the water at one of four starting positions, which were spaced equally around the rim of the pool; each starting position was randomly selected. The latency to reach the platform, swimming distance and swimming speed were measured using the computer-contolled tracking system. Rats that failed to locate the platform within 90 s were guided to the platform manually. On day 19 (i.e., 24 h after the last acquisition trial), the platform was removed and animals underwent the spatial probe trial test. The time spent in the target quadrant and swimming distance were recorded for 90 s. Then, the visible platform test was performed for 3 days after the probe trials. In this section, the visible platform was positioned 1 cm above the water surface. The rats were allowed to locate the platform three times per day. The time to reach the visible platform was recorded and analyzed.

### Step through passive avoidance task

After the water-maze test, rats were tested for non-spatial learning using the step-through passive avoidance (STPA) apparatus (Chinese Academy of Medical Sciences, Beijing, China) [19]. The apparatus consisted of a chamber with two compartments (dark/light), which were connected by a guillotine door. The dark compartment had a stainless steel grid floor available for foot electric shock. In the acquisition trial, all rats were allowed to explore both the compartments for 5 min. 24 h later, each rat was placed in the light compartment for 60 s. Then the guillotine door was raised and the initial latency to enter the dark compartment was recorded. Immediately after the rat entered the dark side, the guillotine door was closed, and an electric shock (0.5 mA, 3 s) was delivered. 5 s later, the rat was removed and returned to its home cage. 24 h after training, the latency was measured following the same procedures in the acquisition trial but no foot shock was delivered; increased latency is indicative of memory enhancement, whereas decreased latency is indicative of memory impairment. The cut-off time was 300 s. Short latencies indicated poorer retention.

### Real-time PCR analysis

Real-time PCR (RT-PCR) was performed according to the manufacturer’s instructions. Total RNA was extracted from 100 mg of hippocampus according to the Trizol protocol (TaKaRa Biotechnology Co., Ltd., Dalian, China). cDNA was prepared by using oligo (dT) as a primer and Moloney murine leukemia virus reverse transcriptase (TaKaRa Biotechnology Co., Ltd.,Dalian, China) as DNA polymerase. The primers were designed using Primer Premier 5.0 (PREMIER Biosoft International, Palo Alto, California, USA) and synthesized by Invitrogen Trading (Shanghai, China) Co., Ltd.. Thermal cycling conditions and sequences of primers and probes for β-actin, APP, BACE1, PS-1, IDE and NEP are shown in Table 1. All real-time PCR reactions were conducted using the SYBR MX 3000P QPCR system (Genetimes Technology, Inc, Shanghai, China). The target gene was normalized to the levels of the β-actin mRNA, which was not changed by any of the treatments. Gene expression levels were calculated as 2^−△△Ct^ values. Each experiment was carried out 3 times.

**Table 1:** Sequences of the primers for the real-time RT-PCR test

Primer	Sequence	Tm (°C)
β-Actin	F: 5’ AGAGGGAAATCGTGCGTGAC 3’	58
	R: 5’CCATACCCAGGAAGGA AGGCT 3’	
APP	F: 5’ AGAGGTCTACCCTGAACTGC 3’	61
	R: 5’ ATCGCTTACAAACTCACCAACT 3’	
BACE1	F: 5’ CGGGAGTGGTATTATGAAGTG 3’	57
	R: 5’AGGATGGTGATGCGGAAG 3’	
PS1	F: 5’ TGAGGAGGAAGACGAAGA 3’	60
	R: 5’ CACGGCGACATTGTAGGA 3’	
IDE	F: 5’ AGTCAAAGGGCTGGGTAA 3’	58
	R: 5’ TCTATCAAGTCGGGTCTAA 3’	
NEP	F: 5’ GAAGACCGAAATGACCCA 3’	58
	R: 5’ CGGATGTAGTCCCGTAAA 3’	

### Extraction of the hippocampus tissue

All rats were anesthetized with 10 % chloral hydrate (3.5 ml/kg). They were then decapitated, and the brain was removed immediately. Cortical tissue was removed, and the hippocampus, visible as 2 bean-like structures on either side from the midline line of the brain, was separated out into a 2.0 ml freezing tube for preservation in liquid nitrogen.

### Western blot analysis

Western blot analysis was performed [20]. The brain tissues were homogenized with Radio immunoprecipitation assay (RIPA) lysis buffer containing protease inhibitor cocktail and phosphatase inhibitors (Sigma-Aldrich, USA) and centrifuged at 12,000 × g for 30 min. The samples were separated using sodium dodecyl sulfate-polyacrylamide gel electrophoresis, transferred to polyvinylidene fluoride membranes, and incubated with rabbit APP/BACE1/PS1 (1:1000; Cell Signaling Technology, Danvers, MA, USA), rabbit IDE (1:6000; Abcam, Cambridge, MA, USA), rabbit NEP (1:1000; Santa Cruz Biotechnology, Santa Cruz, CA, USA), rabbit anti-GAPDH (1:1000; Cell Signaling Technology, USA) antibodies overnight at 4 °C. The membranes were blocked with non-fat milk for 1h at room temperature, then incubated with goat anti-rabbit IgG horseradish peroxidase (HRP) (1:2000; Cell Signaling Technology, USA) for 120 min. The bands were visualized using a Kodak Digital Science ID and quantified with Image J software 6.0.

### Statistical analysis

Repeated measures ANOVA was used to analyze the escape latencies in the MWM test. Other data were analyzed by one-way ANOVA followed by LSD or Dunnett’s comparison test.. Differences between groups were considered significant when *P* < 0.05. Data are expressed as mean ± SEM.

## Results

### Effect of CDS on Aβ_25–35_-induced memory deficit in the Morris water maze test

**Fig. 2:**
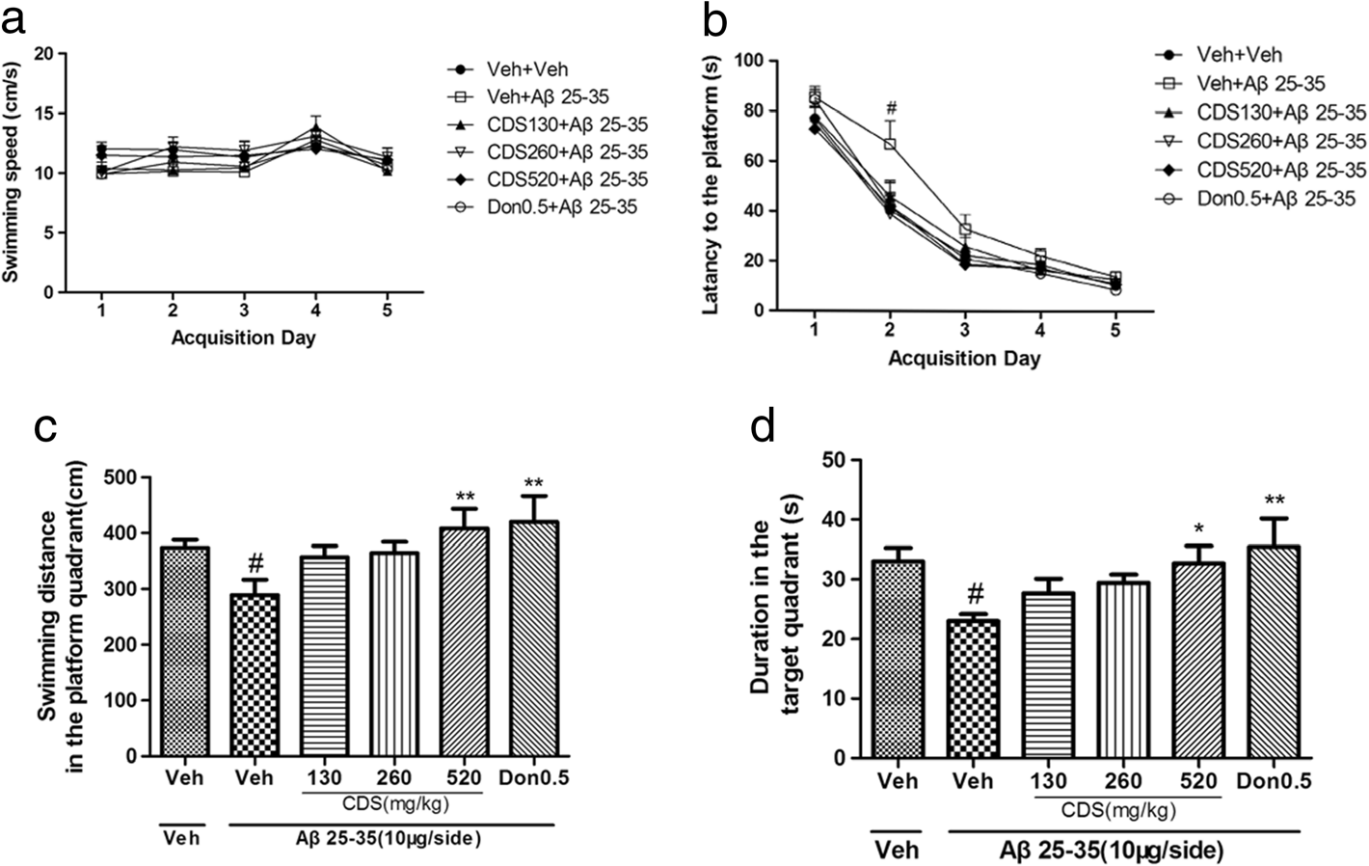
Effect of CDS on Aβ_25–35_-induced memory deficit in the Morris water maze. **a** The swimming speed did not differ between treatment groups over time. **b** Changes in escape latency to the platform during acquisition trials in rats treated with vehicle(Veh), Aβ_25–35_ + Veh, Aβ_25–35_ + CDS (130 mg/kg, 260 mg/kg and 520 mg/kg) or Aβ_25–35_ + Donepezil (0.5 mg/kg). Aβ_25–35_ (10 μg/side) significantly increased escape latency for 2 days relative to other groups. **c**, **d**. Effects of Aβ_25–35_ with or without treatment on the swimming distance (**c**) and duration (**d**) in the target quadrant in the probe trial performed 24 h after the last training trial. Aβ_25–35_-treated rats displayed decreases in both the swimming distance and duration; these were reversed by CDS (520 mg/kg) and donpezil. Values shown are means ± SD. *n* = 8 ~ 9; ^#^ *P* < 0.05 vs. Veh alone; ^*^ *P* < 0.05, ^**^ *P* < 0.01 vs. Aβ_25–35_ + Veh

To evaluate the effect of CDS on Aβ_25–35_-induced spatial memory deficit, we examined memory performance of rats treated with CDS, Donepezil and vehicle in the Morris water maze test. As shown in Fig. 2a, two-way repeated measures ANOVA revealed that no significant differences were identified in the swimming speeds during the 5-day acquisition trials in the water maze test (*P* > 0.05). This result confirmed that the sport ability of the rats did not influence the assessment of the test.. A two-way repeated measures ANOVA revealed that all the rats displayed progressive decrease in escape latency to reach the hidden platform [drug effect: F(5,42) = 11.37, *P* < 0.01; day effect: F(4168) = 196.02, *P* < 0.001; group × day interaction: F(20,168 = 1.06, *P* > 0.05)]. *Post-hoc* LSD comparison suggested that Aβ_25–35_-treated rats spent more time locating the platform than the sham-operated groups on day 2 (*P* < 0.05) (Fig. 2b). 24 h after the last acquisition trial, spatial memory of all rats were evaluated by the probe trial test, during which the platform was removed. As shown in Fig. 2b & c, there were significant overall group difference in the druation (F(5,42) = 2.605, *P* < 0.05) and distance (F(5,42) = 2.585, *P* < 0.05) spent in the target quadrant among the six groups. Compared to the sham-operated groups, Aβ_25–35_ decreased both swimming distance (*P* < 0.01, Fig. 2c) and duration (*P* < 0.05; Fig. 2d) in the platform quadrant by LSD comparision. CDS reversed memory deficit induced by Aβ_25–35_ in a dose-dependent manner. CDS at dose of 520 mg/kg and donepezil significantly increased the duration in the platform quadrant (*P* < 0.05 and *P* < 0.01, respectively), as well the swimming distance (*P* < 0.01). Together, these results suggests that CDS has a positive effect on the spatial memory.

### Effect of CDS on Aβ_25–35_-induced memory impairment in the passive avoidance test

**Fig. 3:**
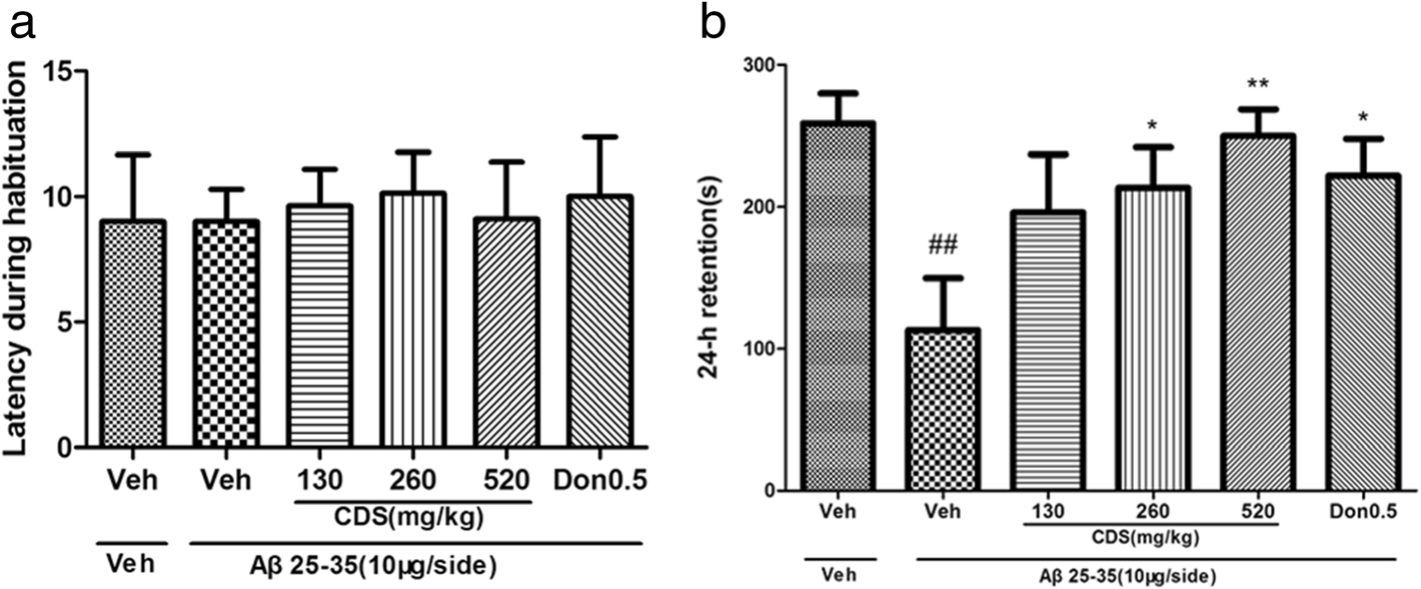
Effects of CDS on the latency to the dark compartment (**a**) during habituation (**b**) 24 h retention after habituation in the step-through passive avoidance test in rats. While the treatment did not alter the latency to enter the dark compartment during habituation, treatment with Aβ_25–35_ decreased the latency after 24 h. CDS reversed the 24 h retention caused by Aβ_25–35_ in a dose-dependent manner. Values shown are means ± SD. *n* = 8 ~ 9; ^#^ *P* < 0.05 vs. Veh alone; *P < 0.05, **P < 0.01 vs. Aβ_25–35_ + Veh

In the step-through passive avoidance task, all the rats took 12 s in average to enter the dark compartment, and the latency did not differ between groups (F(5,42) = 0.063, *P* > 0.05) (Fig. 3a) as analysed by one way ANOVA. In contrast, 24 h after initial training with foot-shock experience, the sham-operated rats exhibited a robust increase in latency, suggesting that the animals acquired memory of the aversive stimulation associated with the darkened compartment. One way ANOVA indicated that there was significant group effect on the step-through latency (F = 3.175, *P* < 0.05). The mean latency of the Aβ-treated rats with or without CDS treatment was significantly changed in the retention test (Fig. 3b). Post hoc LSD tests revealed that model animals treated with vehicle displayed shorter latency compared to the sham group (*P* < 0.01), indicating that Aβ_25–35_ impaired long-term memory in rats. This memory impairment was markedly attenuated by CDS at doses of 260 or 520 mg/kg in a dose-dependent manner (*P* < 0.05 and *P* < 0.01, respectively). Together, results of the passive avoidance test further demonstrated CDS could ameliorate learning and memory impairment of rats, which was caused by Aβ peptide.

### Effect of CDS on mRNA level of APP, BACE1, PS1, IDE and NEP in the hippocampus of rats

**Fig. 4:**
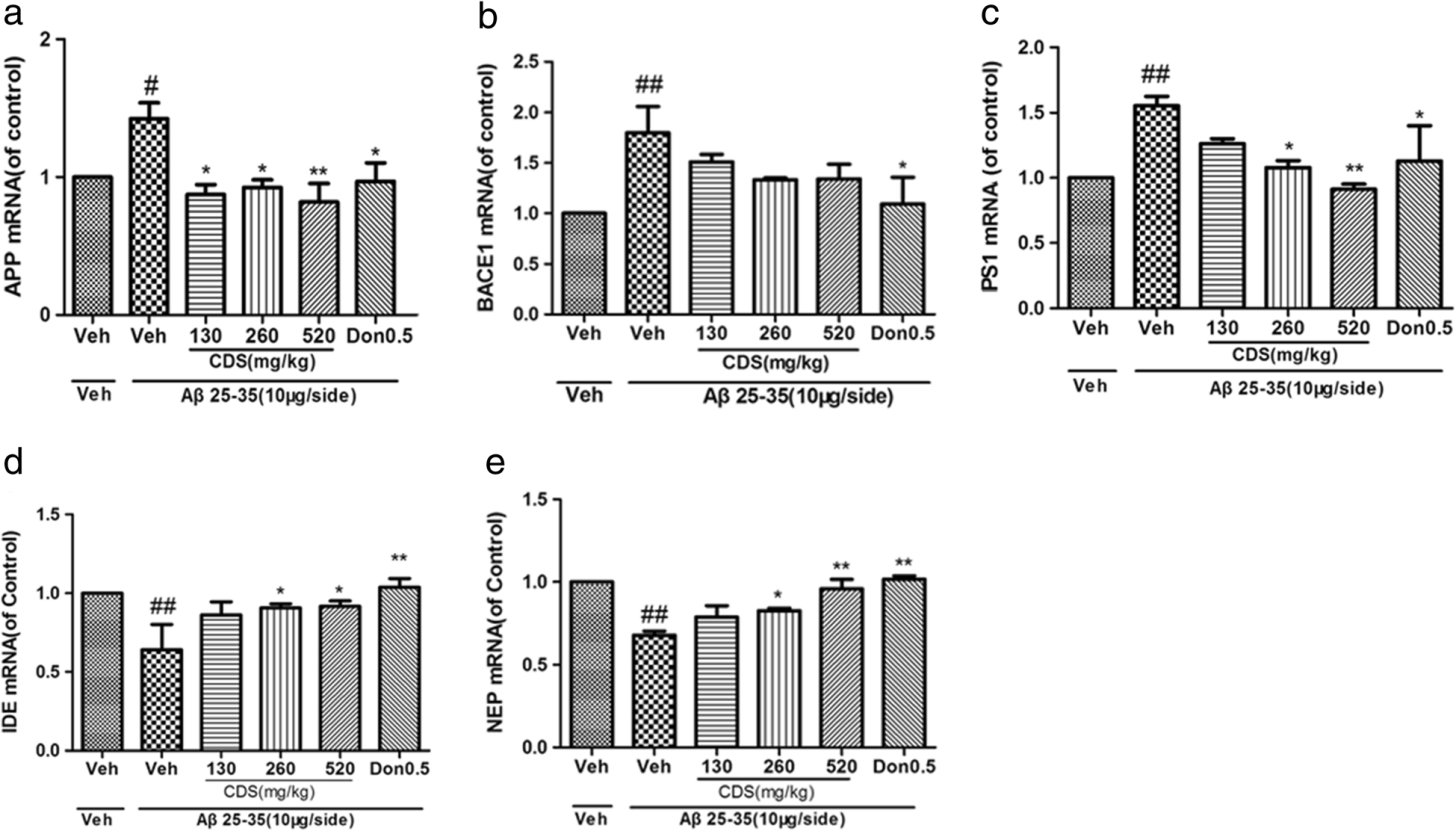
Effects of CDS on mRNA levels of (**a**) APP, (**b**) BACE1, (**c**) PS1, (**d**) IDE and (**e**) NEP in the hippocampus of rats. After 23 days treatment, all the rats were decapitated, then hippocampus was extracted for Real-time RT-PCR. Values shown are means ± SD. n = 3 ~ 4; ^#^ *P* < 0.05, ^##^ *P* < 0.01 vs. vehicle alone; ^*^ *P* < 0.05, ^**^ *P* < 0.01 vs. Aβ_25–35_ + vehicle

To confirm the effect of CDS on the associated enzymes of Aβ production and clearance, we performed Real-time PCR analysis. The results in Fig. 4a-c showed that APP, BACE1 and PS1 mRNA levels in Aβ_25–35_-treated group were significantly increased compared with sham-operated group (*P* < 0.01), whereas CDS treatment significantly reversed increase of APP at doses of 130 mg/kg, 260 mg/kg and 520 mg/kg (*P* < 0.05, *P* < 0.05, *P* < 0.01, respectively) and PS1 at doses of 260 mg/kg and 520 mg/kg (*P* < 0.05, *P* < 0.01, respectively) rather than BACE-1 compared with Aβ_25–35_-treated rats. In addition, in donepezil-treated rats there were significantly reversal in the increase of APP, BACE1 and PS1 mRNA by Aβ_25–35_ (*P* < 0.05).

Rats of model groups showed a significant decline in the mRNA levels of IDE (Fig. 4d, *P* < 0.01) and NEP (Fig. 4e, *P* < 0.01). Interestingly, after 23 days of treatment, CDS increased both IDE and NEP levels in a dose-dependent manner. As shown in Fig. 4d and e, we found that CDS at doses of 260 mg/kg and 520 mg/kg, significantly reversed the decrease of IDE (*P* < 0.05 and *P* < 0.05, respectively) and NEP (*P* < 0.05 and *P* < 0.01, respectively), induced by Aβ_25–35_.

Together, these results indicates that CDS might ameliorate learning and memory ability of rats through decreasing APP, PS1 and increasing IDE, NEP at gene levels.

### Effect of CDS on protein level of APP, PS1, IDE and NEP in the hippocampus of rat

**Fig. 5:**
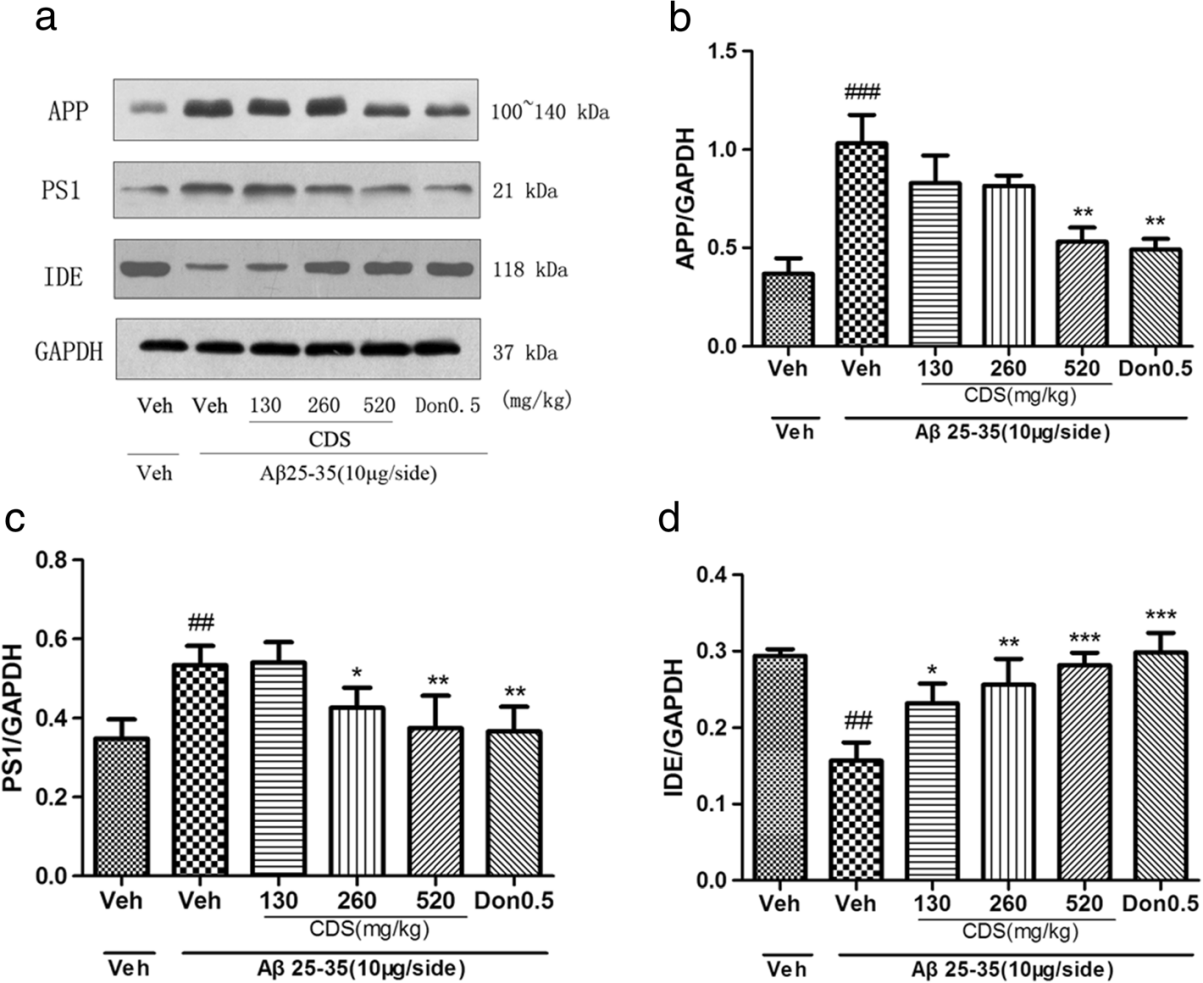
Effect of CDS on protein expression of APP, PS1 and IDE in the hippocampus of rat. **a** Representative band of protein expression and (**b**) quantitative expression levels of APP, (**c**) PS1 and (**d**) IDE. Quantified results were normalized to GAPDH. Values are expressed as mean ± SD (*n* = 3 rats/group). For statistical significance, ^#^ *P* < 0.05, ^###^ *P* < 0.001 compared to sham group, ^*^ *P* < 0.05, ^**^ *P* < 0.01 compared to Aβ_25–35_-treated rats

Given that CDS had significant effects on the mRNA expression of APP, PS1, IDE and NEP, we performed Western blot analysis to confirm its treatment on protein expression. As showed in Fig. 5, consistent with the changes in mRNA levels as described above, protein expression of APP and PS1 in Aβ_25–35_-treated rats significantly increased when compared to the sham group (*P* < 0.05, Fig. 5a, b, c). CDS significantly reversed these changes at a dose of 520 mg/kg. In contrast, Aβ_25–35_-treated rats displayed a significant decreased of IDE expression compared to sham group (*P* < 0.05, Fig. 5d). CDS reversed IDE reduction significantly at three doses. However, we did not find a significant effect of CDS on NEP expression (data not shown).

## Discussion

The treatment of AD is a clinical challenge. The cholinesterase inhibitors like donepezil, rivastigmine and galantamine, N-methyl-d-aspartate antagonist, memantine, are the only agents currently approved by the FDA for the treatment of AD [21]. CDS has been proved to be a potential drug for Alzheimer’s disease in our previous studies [12–15]. The present results revealed the important cognition-enhancing effects of CDS against i.c.v. Aβ_25–35_-induced memory impairment. In Morris water maze, no significant difference in swimming speeds were observed in either acquisition trials or probe test in the present study. This excludes the possibility that the sport ability per se may have contributed to changes in the task in vehicle-, CDS- and donepezil-treated rats. During acquisition trials, all the rats displayed a declined tendency in escape latency. Only on the day 2, the rats of model group took a little more time to reach the submerge platform. However, CDS and donepezil significantly reversed the Aβ_25–35_-induced less time in the target quardrant in the probe test. Most importantly, behavioral test demonstrated that CDS could improve the spatial memory rather than spatial learning. Furthermore, the visible platform test for 3 days after the probe trial showed that all the rats displayed the similar escape latencies to the platform (data not shown), which excluded the influence of visual ability of rats. To further determine the effect of CDS on the memory retention, we performed the passive avoidance task. Avoidance task are comparable to cued and contextual conditioning, which is a fear condition task that measures the ability of an animal to learn and remember the association between an aversive stimulus and environmental cues. In the present study, after 3 s of electric foot shock, AD rats took less time to enter the dark compartment, while treatment with CDS did keep the rats stay more time in the light one. These results indicated that CDS was beneficial to memory retention.

It has long been believed that the accumulation of Aβ is a major neuropathological feature of AD, which is thought to result from an imbalance between Aβ production and clearance [22]. Although CDS can improve learning and memory ability in Aβ_25–35_-induced AD model, its mechanism is remained to be further determined. Aβ protein is, in general, processed through proteolytic cleavage by the β- and γ-secretases from APP [23]. BACE1, known as a member of β-secretases, is an important enzyme that cleaved APP to produce Aβ. PS1 is one of the four core proteins in presenilin complex, which mediate the regulated proteolytic events of several proteins in the cell, including γ-secretase. BACE1 and PS1 up-regulation may play a key role in accumulation of Aβ [24, 25]. An increasing number of studies have focused on inhibiting the production of Aβ and therapeutic strategies aimed to decreased APP, BACE1 and PS1. In addition to Aβ production, recent studies have paid more attention to how Aβ was normally degraded or cleared. Among the Aβ degrading enzymes, IDE and NEP are thought to be most potent. Impairment of IDE and NEP function could also lead to the elevation of Aβ level and therefore contribute to the AD development [26–28]. Interestingly, Qin et. al found that CDS was able to decrease APP mRNA expression in AD rats. Thus, our study was carried out to evaluate the effect of CDS on the associated molecules of production or clearance of Aβ in Aβ_25–35_-induced model rats of AD.

In agreement with previous study, we confirmed that CDS could down-regulate APP expression induced by Aβ in both mRNA and protein levels. Interestingly, we demonstrated for the first time that CDS also had an influence on the mRNA expression of PS1, IDE and NEP except BACE1. Presenilin-1 is one of the most important cleaving enzymes of γ-secrectases, which mainly mediates the regulated proteolytic events of APP [29]. PS-1 mutation can lead to the familiar Alzheimer’s disease. In the present study, similar to APP, we found that CDS also down-regulated PS1 mRNA expression. The results of Western blotting further supported that CDS decreased PS1 expression in protein level, which was caused by Aβ. NEP is able to degrade monomeric and oligomeric form of Aβ [30]. Exogenous injection of recombinant soluble neprilysin reduced Aβ levels and improved learning and memory ability of AD mice [31]. Aβ40 and Aβ42 levels in the brain of NEP knock-out mice were found to increase markedly [32, 33]. Deletion of IDE gene in mice also causes an increase of Aβ levels. APP/PS1 transgene mice model suggested that up-regulation of the IDE and possibly of NEP expression in response to the Aβ accumulation [34]. The present findings showed that i.c.v. injection of Aβ_25–35_ could markedly decrease expression of IDE and NEP, but CDS-treated reversed this phenomenon in a dose-dependent manner in mRNA level. However, CDS only increased IDE expression in protein level rather than NEP. Taken together, we speculate that CDS may exert cognition-enhancing effect via decreasing APP, PS1 level and increasing IDE level.

## Conclusion

In summary, CDS has a positive effect on memory improvement. Data from this study compensated previous studies by supporting that CDS reverses the associated molecular changes caused by Aβ. CDS improved spatial learning and memory by down-regulating APP, PS1 levels and up-regulating IDE levels. Although the mechanism of CDS mediated cognitive-enhancing remains to be elucidated, it is possible that CDS may ameliorate memory function through balancing the production and clearance of Aβ. In future, CDS may have significant therapeutic potential in the treatment of AD patients.

## Abbreviations

APP: Amyloid precursor protein
BACE-1: Beta-site amyloid precursor protein enzyme-1
PS1: Presenilin-1
IDE: Insulin-degrading enzyme
NEP: Neprilysin
AD: Alzheimer’s disease
CDS: Compound danshen
Aβ: Amyloid-beta
MWM: Morris water maze
STPA: Step through passive avoidance
PCR: Polymerase chain reaction
